# Taohuajing reduces oxidative stress and inflammation in diabetic cardiomyopathy through the sirtuin 1/nucleotide-binding oligomerization domain-like receptor protein 3 pathway

**DOI:** 10.1186/s12906-021-03218-0

**Published:** 2021-02-26

**Authors:** Rui Yao, Yu Cao, Changming Wang, Lu Xu, Xuan Zhang, Yuqing Deng, Feng Li, Siwang Wang

**Affiliations:** 1 Department of Traditional Chinese Medicine, the First Affiliated Hospital, the Air Force Medical University, 127 Changle West Road, Xi’an, 710032 Shanxi China; 2 Department of Chinese Materia Medica and Natural Medicines, School of Pharmacy, the Air Force Medical University, 169 Changle West Road, Xi’an, 710032 Shanxi China; 3 Xi’an Hemoqi Medical Technology Co. Ltd., Xi’an, 710075 Shaanxi China

**Keywords:** Taohuajing, Oxidative stress, Inflammation, Diabetic cardiomyopathy, Sirtuin 1, NLRP3

## Abstract

**Background:**

Oxidative stress and inflammation promote the development of diabetic cardiomyopathy (DCM). Therefore, inhibiting these processes may show beneficial effects in the treatment of patients with DCM. Taohuajing (THJ) is prepared using *Persicae semen* (Taoren), *Polygonatum sibiricum* (Huangjing), and *Carthami flos* (Honghua) and may have applications in the treatment of DCM. However, the protective effects of THJ have not been thoroughly assessed. Accordingly, in this study, we aimed to investigate the protective effects of THJ in a model of DCM and further clarify the potential mechanisms.

**Methods:**

A type 2 diabetes mellitus model was generated using male C57BL/6 mice. Echocardiography and histopathology were used to evaluate cardiac function. The expression levels of cytokines were measured using enzyme-linked immunosorbent assays. Western blotting and small interfering RNA were used to evaluate the targets of THJ.

**Results:**

Compared with the control group, DCM mice showed cardiac dysfunction, metabolic disorder, fibrosis, and disorganized ultrastructure, and THJ treatment significantly inhibited these changes significantly. THJ treatment also inhibited the production of reactive oxygen species (ROS) and malondialdehyde (MDA), induced the production of glutathione peroxidase (GSH-Px) and superoxide dismutase (SOD), decreased the levels of pro-inflammatory cytokines, and suppressed the activation of the nucleotide-binding oligomerization domain-like receptor protein 3 (NLRP3) inflammasome. These protective effects were abolished by sirtinol, an inhibitor of sirtuin1 (SIRT1).

**Conclusions:**

Overall, THJ protected the heart from hyperglycemia-induced oxidative stress and inflammation in DCM mice via a mechanism involving SIRT1-mediated antioxidant proteins and suppression of the NLRP3 inflammasome.

## Background

The incidence of type 2 diabetes mellitus (T2DM) has been increasing worldwide as the rates of obesity, sedentary lifestyle, and environmental alterations have increased; accordingly, it is estimated that half a billion people worldwide will be diagnosed with T2DM by 2030 [1]. Chronic diabetes impairs ventricular function, including macrovascular and microvascular, thereby inducing diabetic cardiomyopathy (DCM), which is one of the main causes of death in patients with T2DM [2, 3]. In the clinical setting, the goals of treatment include controlling glucose and lipid levels, managing hypertension, and blocking the renin-angiotensin system; however, controlling these parameters alone cannot prevent the pathological changes associated with DCM [3]. Therefore, it is necessary to develop an effective drug that targets multiple pathologies of DCM in order to prevent or delay the development and progression of DCM.

Several mechanisms are likely to contribute to the development of DCM, including cardiac oxidative stress, inflammation, apoptosis, fibrosis, calcium overload, and mitochondrial dysfunction [4, 5]. The two key metabolic abnormalities, i.e., hyperglycemia and hyperlipidemia, alter the cellular redox status, induce reactive oxygen species (ROS) accumulation, decrease antioxidant protein activities, and disrupt membrane function, resulting in contractile dysfunction within weeks [6–10]. Oxidative stress and inflammation induce cardiac dysfunction, impair insulin signaling, cause heart dysfunction, and decrease endogenous antioxidant levels [11, 12]. Nucleotide-binding oligomerization domain-like receptor proteins (NLRPs), which form a type of inflammasome, have been found to play important roles in the inflammatory process of DCM and other diabetic complications, such as diabetic retinopathy [13, 14]. Overproduction of ROS in cells will activate the NLRP3 inflammasome and increase inflammatory damage [15]. Thus, drugs or chemicals that can inhibit both oxidative stress and inflammation may have beneficial effects in treating DCM.

Sirtuin 1 (SIRT1) belongs to the sirtuin family and plays important roles in regulating inflammation, oxidative stress, apoptosis, and the cell cycle by deacetylating chromatin histones and multiple transcription factors, including Forkhead transcription factors (FOXOs) and nuclear factor (NF)-κB [16–18]. FOXO3a, a FOXO family member expressed in the heart, transcriptionally regulates the expression of manganese superoxide dismutase (MnSOD), which protects cells from oxidative stress [19]. Additionally, SIRT1 interacts with NF-κB by inhibiting the transcription of p65, thereby blocking the expression of inflammatory factors [20, 21]. Accordingly, regulating the expression and/or the activity of SIRT1 may be useful to manage inflammation and oxidative stress induced by hyperglycemia and hyperlipidemia in DCM.

Traditional Chinese medicine (TCM), which is based on ancient philosophies regarding the use of herbs and plants, has been shown to be effective in treating DCM, with fewer side effects [22, 23]. Taohuajing (THJ) is composed of *Persicae semen* (*Prunus persica (L.) Batsch*, Taoren), *Carthami flos* (*safflower, Carthamus tinctorius (L.)*, Honghua), and *Polygonatum sibiricum(F.)* (Huangjing), based on the TCM theory. The pair of *Persicae semen* and *Carthami flos* is a classical medicinal pair used in many well-known prescriptions, including Taohongsiwutang, Buyanghuanwutang, and Taorenchengqitang [24, 25]. Extracts of *Carthami flos* have antidiabetic effects in an alloxan-induced diabetic model in rats and exhibit antioxidative effects in scavenging free radicals and enhancing endogenous antioxidant activity in rat cardiac microvascular endothelial cells [26, 27]. Moreover, *Persicae semen* can inhibit macrovascular fibrosis by regulating the AKT pathway in streptozotocin (STZ)-induced diabetic rats [28]. *Polygonatum sibiricum* has many physiological effects, including antioxidant, anti-aging, anti-inflammatory, hypolipidemic, and heart protective effects, and has been used in the treatment of diabetes mellitus, exhibiting excellent antidiabetic activity [29–31]. The total saponins from *Polygonatum sibiricum* can promote glycogenesis and glucose utilization in peripheral tissue, thereby controlling blood glucose levels [32]. Additionally, polysaccharides from *Polygonatum sibiricum* also show protective effects by inhibiting the binding of glycosylated end-products in cardiac tissues in diabetic model mice [33].

Although these individual medicines have been shown to have antidiabetic activities and cardioprotective effects, no studies have reported whether the combination of these three medicines can be used to treat diabetic cardiomyopathy. Therefore, in the current study, we assessed the protective effects of THJ on a DCM model induced by a high-fat diet (HFD) and STZ.

## Methods

### Materials

*Persicae semen* (Taoren) was obtained from Hebei province, China. *Carthami flos* (Honghua) was purchased from Xinjiang province, China. *Polygonatum sibiricum* (Huangjing) was purchased from Shaanxi, China. 2,3,5-Triphenyltetrazolium chloride (TTC) was purchased from Sigma-Aldrich (St. Louis, MO, USA). Kits for the detection of insulin, SOD, malondialdehyde (MDA), glutathione peroxidase (GSH-Px), and caspase 3 were obtained from Nanjing Jiancheng Bioengineering Institute (Nanjing, China). Dihydroethidium (DHE) was obtained from Invitrogen (CA, USA). The primary antibodies against NPL65 (cat. no. 13158), cleaved-caspase 3 (cat. no. 9664), and cleaved-caspase 9 (cat. no. 20750) were obtained from Cell Signaling Technologies (MA, USA). The NAD+/NADH assay kit was purchased from Abcam (Carlsbad, CA, USA). Anti-acetylated (Ac)-FOXO3 and anti-SIRT1 primary antibodies were obtained from Santa Cruz Biotechnology (Santa Cruz, CA, USA). All other materials used in this study were commercially available.

### Preparation of THJ

*Persicae semen* (300 g), *Carthami flos* (180 g), and *Polygonatum sibiricum* (300 g) were obtained (The weight ratio of *Persicae semen*: *Carthami flos*: *Polygonatum sibiricum* is 5:3:5*)* and crushed into small pieces, and the concoction extracted with deionized water (1:10, w/v) twice for 30 min each. Filtrates from the two batches were combined, concentrated, and spray dried.

### Animals

C57BL/6 mice (8–10 weeks old, 23–25 g) were purchased from the Animal Experimental Center of the Air Force Medical University. Mice were housed with free access to water and standard food in an experimental animal room with a 12/12-h light/dark cycle, temperature maintained at 22 ± 2 °C, and relative humidity of 61–65%. The animals were provided with a standard laboratory diet and water and were checked every day for any health issues. All protocols in this study were approved by the Ethics Committee for Animal Experimentation of the Air Force Medical University and performed according to the National Institute of Health Guide for the Care and Use of Laboratory Animals (NIH Publications No. 80–23) revised in 1996 and the Animal Research Reporting In Vivo Experiments guidelines.

### DCM model and groups

After acclimation for 1 week, mice in the diabetes groups were fed an HFD containing 45% kcal from fat for 4 weeks and then intraperitoneally injected with 60 mg/kg STZ (Sigma) dissolved in citrate buffer (pH 4.5) for 3 days. The same volume of citrate buffer was intraperitoneally injected into mice in the control group. After 2 weeks, fasting-blood glucose (FBG) was measured using a BREEZE2 meter (Bayer, Mishawaka, IN, USA). Mice with an FBG of more than 11.1 mM were considered diabetic and were used in further studies. The DCM model was induced according to previous studies [34]. Animals were randomly assigned to five groups: control group (Con, *n* = 15), DCM group (DCM, *n* = 15), low-dose THJ-treated DCM group (0.125 g/kg/day, THJ-L, n = 15), medium-dose THJ-treated DCM group (0.25 g/kg/day, THJ-M, n = 15), and high-dose THJ-treated DCM group (0.5 g/kg/day, THJ-H, n = 15). Mice in the THJ groups were administered THJ by gavage at doses of 0.125, 0.25, or 0.5 g/kg/day for 12 weeks. Mice in the DCM groups were administered the same volume of vehicle by gavage. Mice in both the DCM and THJ groups were given free access to an HFD, and mice in the Con group were fed standard chow. Because the formula for THJ was modified based on the Tonghongsiwu decoction, the dosages of THJ in animal experiments were set according to the dosage of Tonghongsiwu decoction and our preliminary experiment [35, 36]. After all the experiments were completed, all mice were euthanized with 150 mg/kg pentobarbital sodium through intraperitoneal injection under standard protocols. All efforts were made to minimize suffering.

### Determination of cardiac function

At the end of the experiment, mice were re-anesthetized using 1.0% isoflurane (the heart rate was maintained at 400–500 bpm), and cardiac function was determined by transthoracic echocardiography with a Visual-Sonics Vevo 770 ultrasound system (Toronto, Canada). Left ventricular fractional shorting (LVFS), left ventricular ejection fraction (LVEF), left ventricular end-diastolic volume (LVEDV), and left ventricular end-systolic volume (LVESV) were determined using computer algorithms.

### Hematoxylin-eosin (HE) and Masson’s trichrome staining

After measuring cardiac function, hearts were collected, and 4% paraformaldehyde solution was used to fix the heart tissues. Heart tissues were then embedded in paraffin and sectioned at 5 μm thickness. Sections were incubated with 1% TTC solution (pH 7.4) for 15 min at 37 °C. Extracellular collagen deposition in the heart tissue was examined by Masson’s trichrome staining.

### Determination of biochemical indexes

Blood samples were collected from mice after sacrifice, centrifuged at 2500×*g* for 15 min at 4 °C to collect the supernatants, and stored at − 80 °C. Relative levels of tumor necrosis factor-α (TNF-α), interleukin (IL)-6, GSH-Px, IL-1β, SOD, and MDA were determined using enzyme-linked immunosorbent assay (ELISA) kits, according to the manufacturer’s protocols. Insulin levels were measured using an insulin assay kit, and the insulin sensitivity index (ISI) was calculated as 1/(blood glucose × serum insulin). NAD+ and NADH levels were measured using an NAD+/NADH assay kit, and the NAD+/NADH ratio was calculated.

### Transmission electron microscopy (TEM) analyses of heart tissues

TEM was used to detect changes in the ultrastructure morphology of myofibrils and mitochondria. Tissues with a thickness of 60–64 nm and pieces measuring 1 mm^3^ were cut from the left ventricle of mouse heart tissue, and sections were fixed with 2.5% glutaraldehyde (pH 7.4) for 2 h. Sections were dehydrated in alcohol, embedded, polymerized, stained with uranyl acetate and lead citrate, and then observed by TEM (JEM-1200EX; Japan).

### ROS detection

ROS in myocardial frozen sections was detected by DHE staining, as previously reported [37, 38]. After staining with DHE and 4′,6-diamidino-2-phenylindole, tissue sections were observed using an Olympus FV1000 laser confocal microscope (Olympus, Japan). The fluorescence density value was determined using an Image-Pro Plus (Version 6.0, Media Cybernetics, USA).

### Western blotting

RIPA lysis buffer was prepared with 1% phenylmethylsulfonyl fluoride and 1% protease inhibitor cocktail. Heart tissues were collected, rinsed, homogenized with RIPA lysis buffer at 4 °C for 30 min, and centrifuged to collect supernatants. Protein concentrations were detected using a BCA protein assay kit (Nanjing Jiancheng) according to the manufacturer’s instructions. Total proteins (30 μg) were loaded and separated by sodium dodecyl sulfate polyacrylamide gel electrophoresis on 10% gels. Polyvinylidene difluoride membranes were activated with methyl alcohol, and the proteins were then transferred onto the membranes. Next, the membranes were blocked with 5% nonfat milk for 1 h at 37 °C. After washing, the membranes were incubated with primary antibodies targeting NLRP3, thioredoxin interacting protein (TXNIP), caspase 1 p20, IL-1β p17, apoptosis-associated speck-like protein containing a CARD (ASC), SIRT1, Ac-FOXO3a, Ac-SOD2 overnight at 4 °C. After rinsing and incubating with secondary antibodies at 37 °C for 1 h, the bands were detected using enhanced chemiluminescence (Pierce). The bands were scanned using a Bio-Rad Chemidoc XR+ Gel Imaging System, and the optical densities were quantified using a Bio-Rad Image Analysis System. β-Actin was used as the internal control.

### Statistical analysis

Statistical analysis was performed using Graph Pad Prism 5.0. Values are shown as means ± standard deviations from at least three different experiments. Differences among experimental groups were analyzed by one-way analysis of variance, followed by Tukey’s multiple comparisons tests. Results with *P* values of less than 0.05 were considered statistically significant.

## Results

### THJ prevented systemic metabolic abnormities in diabetic mice

As shown in Table 1, levels of glucose and lipids (triglycerides [TGs] and total cholesterol [TC]) were both significantly increased in diabetic mice (*P* < 0.05). Additionally, the HFD and STZ treatment significantly decreased insulin levels in the blood (*P* < 0.05) and impaired insulin sensitivity, indicating that diabetes induced systemic metabolic abnormities in diabetic mice. After treatment with THJ, glucose and lipid levels were decreased, and insulin sensitivity was improved in a dose-dependent manner. Notably, 0.5 g/kg THJ showed the best effects. These results indicated that THJ alleviated the systemic metabolic abnormalities induced by HFD and STZ treatment in diabetic mice.

**Table 1:** Effects of THJ on systemic metabolic abnormalities in diabetic mice

	Control	DCM	THJ-L	THJ-M	THJ-H
Blood glucose (mM)	3.67 ± 0.84	24.91 ± 2.71^##^	20.14 ± 1.76	18.32 ± 1.15^*^	18.5 ± 1.08^*^
TG (mM)	0.53 ± 0.07	4.12 ± 0.52^##^	3.81 ± 0.43	3.15 ± 0.38^**^	2.14 ± 0.31^**^
TC (mM)	1.74 ± 0.33	5.13 ± 0.57^##^	4.04 ± 0.51^*^	3.15 ± 0.44^**^	2.47 ± 0.39^**^
INS (mM)	12.72 ± 1.36	7.15 ± 0.87^##^	7.31 ± 0.91	7.7 ± 1.03^*^	8.52 ± 1.15^**^
ISI	0.022 ± 0.003	0.0057 ± 0.00065^##^	0.007 ± 0.0013	0.0076 ± 0.0009^*^	0.0102 ± 0.0024^**^

Data are shown as means ± standard deviations, n = 6–8/group

^##^*P* < 0.01 versus control group, ^*^P < 0.05, ^**^P < 0.01 versus the DCM group

### THJ ameliorated diabetes-induced myocardial dysfunction

**Fig. 1:**
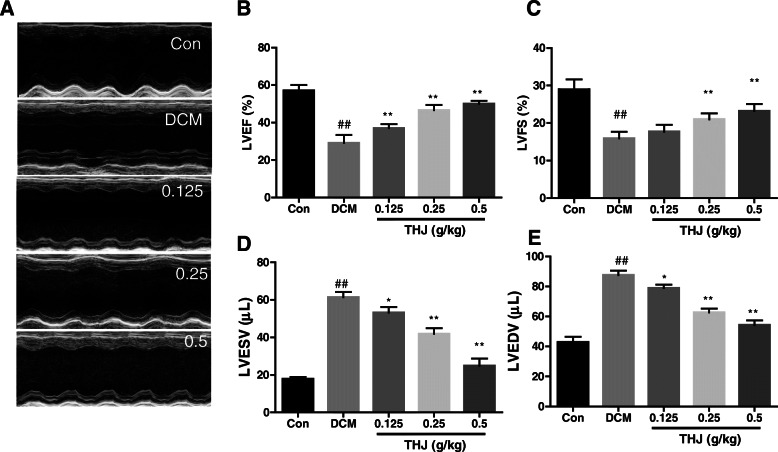
Effects of THJ on cardiac hypertrophy and dysfunction in DCM model mice. **a**. M-mode echocardiography. Representative images were selected from three measurements. Effects of THJ treatment on the cardiac function indicators LVEF (**b**), LVFS (**c**), LVESV (**d**), and LVEDV (**e**). Values are shown as means ± standard deviations (*n* = 6–8). ^##^*P* < 0.01 versus the Con group; ^*^P < 0.05, ^**^P < 0.01 versus the DCM group

To investigate whether THJ protected against diabetes-induced myocardial dysfunction, cardiac function was examined in DCM model mice. LVFS and LVEF, two very important indicators for cardiac function, were recorded by echocardiography. As shown in Fig. 1a–c, compared with control mice, LVEF and LVFS were significantly decreased in the DCM group; however, THJ treatment significantly increased LVEF and LVFS. Additionally, THJ treatment reduced the HFD+STZ-induced increases in LVEDV (Fig. 1d) and LVESV (Fig. 1e) in DCM mice. These results implied that THJ treatment significantly alleviated myocardial dysfunction in DCM mice.

### THJ ameliorated diabetes-induced myocardial dysfunction in DCM model mice

**Fig. 2:**
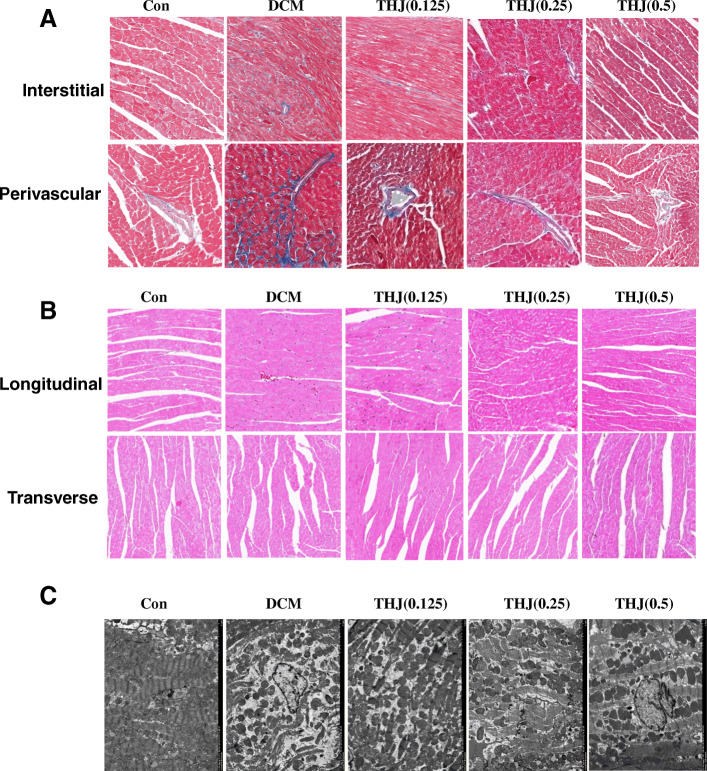
Effects of THJ on pathological changes in myocardial tissues in DCM model mice. **a**. Representative images of Masson’s trichrome staining of interstitial and perivascular tissues. **b**. Representative images of HE staining of longitudinal and transverse sections of heart tissues. **c**. Representative images of TEM

Myocardial dysfunction is partly induced by myocardial fibrosis, which results in disorders of the pathogenic structure and remodeling of hearts. To study whether THJ affected myocardial fibrosis, we carried out Masson staining. As shown in Fig. 2a, compared with the Con group, interstitial and perivascular Masson staining demonstrated that collagens were significantly increased in DCM mouse hearts. Compared with the DCM group, fibrosis and collagen contents were both decreased in interstitial and perivascular areas.

HE staining results showed that there were major pathogenic structural changes in longitudinal and transverse sections, and cardiomyocytes and hypertrophic myocardium were significantly disrupted in the hearts of DCM model mice. In the THJ treatment group, pathological structural changes were markedly alleviated compared with those in the DCM group (Fig. 2b). TEM was also used to observe microstructure changes. In control mice, typical symmetric myofibrils, well-organized sarcomeres, and mitochondria were observed (Fig. 2c). However, tissues from DCM hearts showed serious injury to the left ventricular ultrastructure, including ruptured and irregular myofibrils, swollen mitochondria, and coalescence of irregular mitochondria. In the THJ treatment groups, these changes were significantly alleviated, and the cardiac ultrastructure in the high-dose treatment group was similar to that in the normal group.

### THJ ameliorated diabetes-induced oxidative stress in DCM model mice

**Fig. 3:**
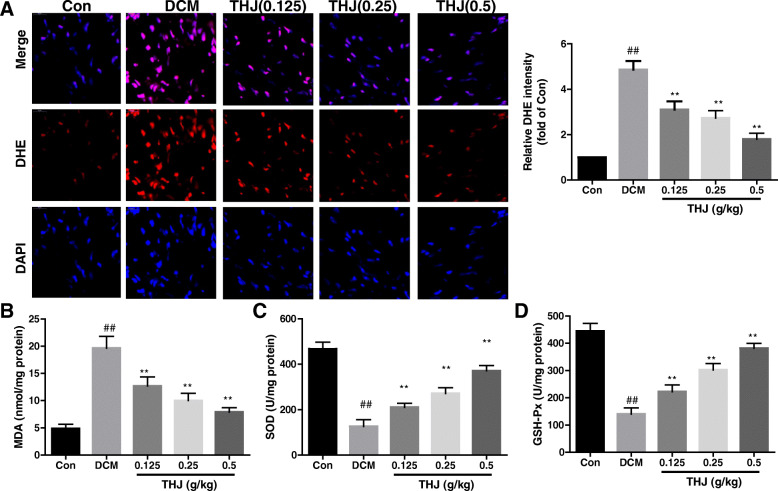
Effects of THJ on oxidative stress in DCM model mice. **a**. DHE staining images from three measurements are shown. Statistical results of DHE intensity are given in the lower panel. **b**. Effects of THJ on MDA levels in myocardial tissues. **c**. Effects of THJ on SOD activities in myocardial tissues. **d**. Effects of THJ on GSH-Px activities in myocardial tissues. Values are showed as means ± standard deviations (*n* = 6–8). ^##^*P* < 0.01 versus the Con group, ^**^P < 0.01 versus the DCM group

ROS levels in heart tissues were detected by DHE staining. As shown in Fig. 3a and b, compared with the Con group, the intensity of DHE fluorescence was significantly increased in the DCM group. Treatment with THJ significantly deceased the intensity of DHE fluorescence, indicating that THJ inhibited the generation of ROS in the heart. Moreover, MDA levels were significantly elevated in the DCM group and decreased in the THJ treatment groups. Notably, the enzymatic activities of SOD and GSH-Px were significantly decreased in the DCM group (Fig. 3c and d). However, THJ significantly elevated the enzymatic activities of SOD (Fig. 3c, *P* < 0.01) and GSH-Px (Fig. 3d, *P* < 0.01) in a dose-dependent manner. Overall, these results indicated that THJ treatment inhibited oxidative stress in DCM model mice.

### THJ inhibited inflammation and NLRP3 inflammasome activation in DCM model mice

**Fig. 4:**
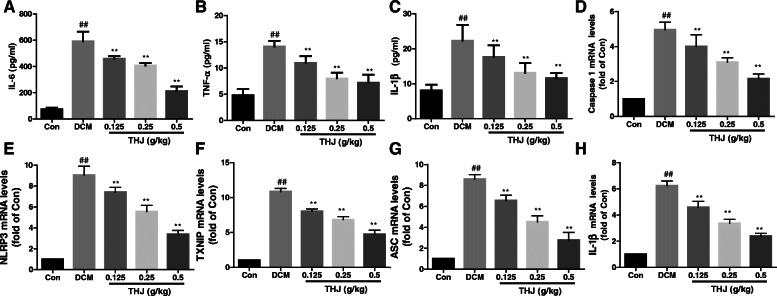
Effects of THJ on the expression levels of inflammatory factors and NLRP3. **a**. Effects of THJ on IL-6 levels in the serum. **b**. Effects of THJ on TNF-α levels in the serum. **c**. Effects of THJ on IL-1β levels in the serum. Effects of THJ on the relative mRNA expression levels of caspase 1 (**d**), *NLRP3* (**e**), *TXNIP* (**f**), *ASC* (**g**), and *IL-1β* (**h**) in myocardial tissues. n = 6–8. ^##^*P* < 0.01 versus the Con group, ^**^P < 0.01 versus the DCM group

Next, to evaluate the inflammatory response in DCM model mice, serum levels of inflammatory cytokines (i.e., TNF-α, IL-6, and IL-1β) were measured. As shown in Fig. 4, the levels of TNF-α (Fig. 4a), IL-6 (Fig. 4b), and IL-1β (Fig. 4c) were significantly increased in the DCM group compared with those in the Con group. In the THJ treatment groups, serum levels of inflammatory cytokines were significantly decreased in a dose-dependent manner.

**Fig. 5:**
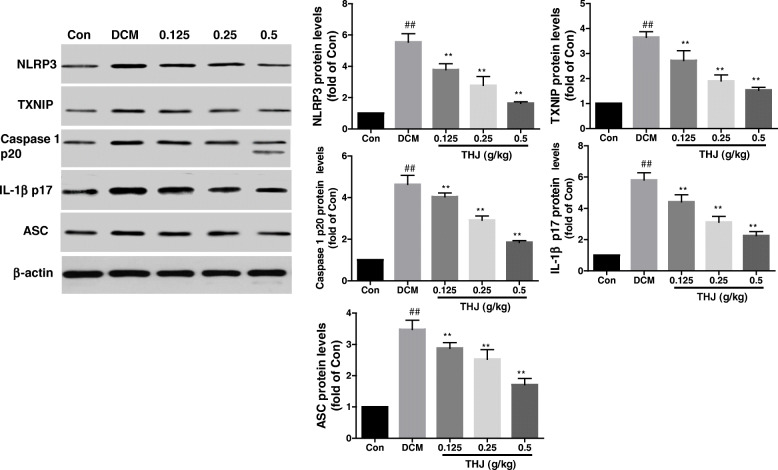
Effects of THJ on the expression levels of NLRP3-related proteins. Representative blots of NLRP3, TXNIP, caspase 1 p20, IL-1β p17, and ASC are shown on the left. Histograms show quantitative expression levels of these targets on the right. Expression levels were normalized to the expression of β-actin. Values are shown as means ± standard deviations from three different experiments. ^##^*P* < 0.01 versus the Con group, ^**^P < 0.01 versus the DCM group

To assess whether the NLRP3 inflammasome was activated by diabetes, the expression levels of NLRP3 and related proteins in left ventricular tissue were measured by polymerase chain reaction (PCR) and western blotting. The mRNA levels of caspase-1 (Fig. 4d), *NLRP3* (Fig. 4e), *TXNIP* (Fig. 4f), *ASC* (Fig. 4g), and *IL-1β* (Fig. 4h) in the DCM group were significantly higher than those in the Con group. Additionally, protein levels of NLRP3 and related proteins were also higher than those in the Con group (Fig. 5). Compared with the DCM group, the mRNA and protein levels of NLRP3 and related proteins (i.e., caspase-1, TXNIP, ASC, and IL-1β) were significantly reduced in the THJ treatment groups.

### THJ inhibited oxidative stress by activating SIRT1

**Fig. 6:**
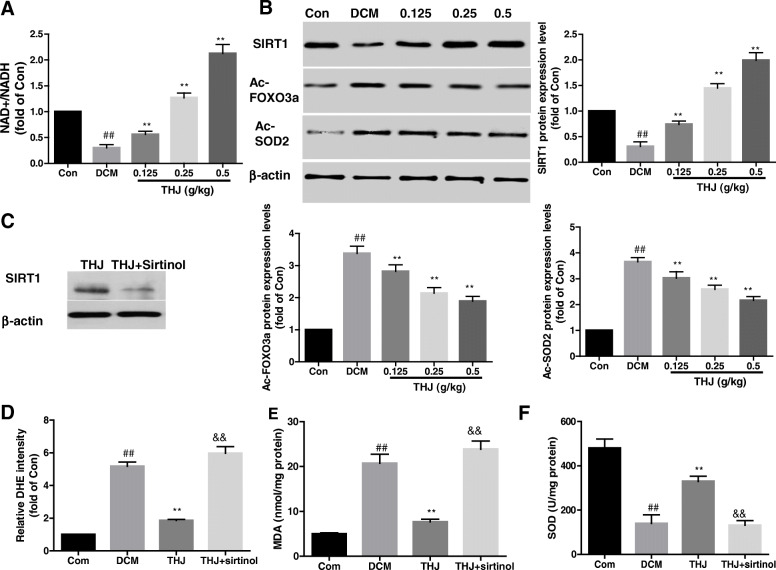
Effects of THJ on the SIRT1 pathway. **a**. Effects of THJ on the NAD+/NADH ratio in myocardial tissues. **b**. Detection of protein expression levels of Ac-SOD2, SIRT1, and Ac- FOXO3a using western blotting. **c**. Mice were treated with the SIRT1 inhibitor sirtinol for 12 weeks and treated with THJ. The effects of THJ on SIRT1 expression levels were assessed. **d**. DHE intensity levels after treatment with the SIRT1 inhibitor sirtinol. **e**. Effects of sirtinol on MDA levels. F. Effects of sirtinol on SOD levels. Values are shown as means ± standard deviations from three different experiments. ^##^*P* < 0.01 versus the Con group, ^**^P < 0.01 versus the DCM group, ^&&^*P* < 0.01 versus the THJ group

Next, we assessed activation of SIRT1 signaling pathways. In the DCM group, the NAD+/NADH ratio was lower than that in the Con group (Fig. 6a), whereas THJ treatment significantly elevated the NAD+/NADH ratio in a dose-dependent manner. SIRT1 and its two main downstream proteins, FOXO3a and SOD2, were detected. As shown in Fig. 6b, SIRT1 expression levels were significantly inhibited, and Ac-SOD2 and Ac-FOXO3a levels were both increased in the DCM group compared with those in the Con group. Interestingly, THJ treatment dramatically reversed these changes compared with the DCM group. To confirm the role of SIRT1 in mediating the effects of THJ treatment, mice were treated with the SIRT1 specific inhibitor sirtinol. As shown in Fig. 6c, sirtinol treatment decreased protein expression levels of SIRT1. Compared with the THJ group, the THJ+sirtinol group showed higher ROS (Fig. 6d) and MDA levels (Fig. 6e) and lower SOD levels (Fig. 6f), indicating that the inhibitory effects of THJ on oxidative stress were abolished by sirtinol.

### Inhibition of SIRT1 suppressed THJ-mediated anti-inflammatory activity

**Fig. 7:**
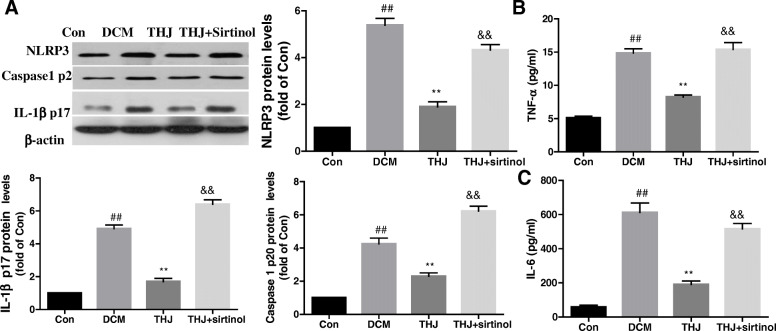
Pharmacological modulation of SIRT1-regulated THJ-induced anti-inflammatory activity. **a**. Mice were treated with the SIRT1 inhibitor sirtinol for 12 weeks and then treated with THJ. Protein expression levels of NLRP3, caspase-1 p20, and IL-1β p17 were evaluated after different treatments. **b**. TNF-α levels in DCM mice. **c**. IL-6 levels in DCM mice. Values are shown as means ± standard deviations from three different experiments. ^##^*P* < 0.01 versus the Con group, ^**^P < 0.01 versus the DCM group, ^&&^*P* < 0.01 versus the THJ group

To further confirm the roles of SIRT1 in THJ-mediated anti-inflammatory activity in DCM model mice, mice were treated with sirtinol for 12 weeks. As shown in Fig. 7a, sirtinol abolished the inhibitory effects of THJ on NLRP3 inflammasome activation compared with that in the THJ group. Additionally, we also found that sirtinol treatment blocked the inhibition of TNF-α and IL-6 expression in DCM model mice (Fig. 7b and c).

## Discussion

T2DM is characterized by hyperglycemia; disruption of carbohydrate, fat, and protein metabolism; and insulin resistance. Collectively, these factors accelerate the development of cardiomyopathy [39]. DCM is one of the most serious complications of advanced T2DM [40] and is characterized by myocardial dilatation and hypertrophy as well as a decrease in the systolic and diastolic functions of the left ventricle. Additionally, DCM can directly lead to cardiac dysfunction and eventually progress to heart diseases, such as heart failure, arrhythmia, and cardiogenic shock [41, 42]. However, therapeutic drugs for DCM are not efficacious in the clinical setting; thus, new medicines or treatment are needed.

TCM has been used for thousands of years and has been shown to be beneficial for preventing and treating DCM with minimum side effects [43, 44]. THJ is a TCM developed from Taohongsiwutang and has been used to treat diabetes and diabetes-related complications [45]. This prescription has putative functions in promoting blood circulation to remove blood stasis and in dredging the channels of humans, based on TCM theory [46–48]. However, the effects of THJ on DCM have not been reported. In this study, we aimed to investigate the effects of THJ on DCM induced by HFD and STZ and to explore the potential mechanisms. We expect the results could provide reference information to promote the rational use of THJ in the clinic.

In this study, we found that blood glucose, TG, and TC levels were elevated, and insulin resistance was induced by HFD and STZ treatment. Blood glucose and lipid levels were both significantly decreased in a dose-dependent manner after THJ treatment for 12 weeks, and insulin resistance was significantly alleviated, indicating that THJ alleviated systemic metabolic disorders in diabetic mice. Additionally, diabetes induced heart dysfunction, myofibril and mitochondrial dysfunction, and cardiac ultrastructure disorganization in DCM model mice. Importantly, THJ treatment significantly ameliorated heart dysfunction and reduced pathological alterations in myofibrils and mitochondria, indicating that THJ protected cardiac muscle from structural changes in DCM model mice.

The main characteristics of diabetes are chronic hyperglycemia and hyperlipidemia, and these two factors contribute to the development of cardiac dysfunction [49]. Increased glucose and lipid metabolism enhances the production of ROS by mitochondria and destroys the antioxidant system, which induces oxidative stress [42]. ROS cause protein, DNA, and RNA damage and induce cardiomyocyte apoptosis, resulting in a reduction in myocardial contractility and leading to myocyte fibrosis [40]. Consistent with previous studies, we found that ROS levels were elevated in DCM heart tissues and that the levels of antioxidant proteins (GSH-Px and SOD) were decreased, whereas MDA levels were increased. However, following treatment with THJ, ROS and MDA levels were reduced, and antioxidant protein levels were elevated in a dose-dependent manner, indicating that THJ protected the heart from oxidative stress in DCM model mice.

In the diabetic state, chronic hyperglycemia induces the secretion of pro-inflammatory cytokines and leads to persistent inflammation, which exacerbates or aggravates myocardial injury [50]. In the current study, the pro-inflammatory cytokines TNF-α, IL-6, and IL-1β were significantly upregulated in DCM model mice. Many studies have shown that the NLRP3 inflammasome can regulate IL-1β activation in diabetes [51–53]. Hyperglycemia activates the NLRP3 inflammasome, accompanied by TXNIP activation, which inhibits the expression of thioredoxin [54]. The NLRP3 inflammasome contains NLRP3, caspase recruitment domain (ASC), and caspase-1 [55]. Once activated by some stimulation, NLRP3 combines with its adaptor ASC, catalyzes pro-caspase-1 to form an active caspase-1 p10/20 tetramer, which processes pro-IL-1β into its mature form, IL-1β [56, 57]. In this study, we demonstrated that the mRNA and protein levels of NLRP3 and related proteins were increased in the DCM group, indicating that the NLRP3 inflammasome was activated in diabetic myocardial tissues. In the THJ treatment groups, the expression levels of NLRP3, TXNIP, caspase-1 p10, and IL-1β p17 were significantly decreased in a dose-dependent manner. Thus, our results indicated that THJ inhibited activation of the NLRP3 inflammasome, which was induced by diabetes.

Antioxidant proteins and NLRP3 are regulated by SIRT1. Therefore, to further understand the antioxidative and anti-inflammatory mechanisms of THJ, we evaluated the roles of SIRT1. SIRT1 plays some important roles in regulating the stress response, such as oxidative stress, inflammation, and apoptosis [58]. SIRT1 induces deacetylation of FOXO3a, thereby increasing the ability of FOXO3a to regulate antioxidant proteins (e.g., SOD and CAT) and DNA repair [59, 60]. Additionally, SIRT1 induces deacetylation of SOD2 directly and increases the antioxidative capacity of SOD2 [61]. In the current study, SIRT1 protein expression levels and downstream proteins (Ac-FOXO3a and Ac-SOD2) were detected to evaluate the regulatory effects of THJ on the SIRT1 pathway. Interestingly, THJ treatment increased the levels of SIRT1, Ac-FOXO3a, and Ac-SOD2 and enhanced the NAD+/NADH ratio, which were decreased in DCM model mice. A previous study showed that SIRT1 has negative regulatory effects on NLRP3 activation in vascular endothelial cells [62]. In this study, we found that SIRT1 expression levels were suppressed by treatment with the SIRT1 inhibitor sirtinol, accompanied by enhanced NLRP3 inflammasome activation and oxidative stress. These results indicated that the mechanisms through which THJ suppressed oxidative stress and the NLRP3 inflammasome were SIRT1 dependent.

## Conclusion

In this study, we demonstrated that THJ protected myocardial tissue from diabetes-induced injury. The beneficial effects of THJ may be dependent on reducing ROS production and inhibiting NLRP3 activation, thereby blocking excessive secretion of pro-inflammatory cytokines, and further studies indicated that these protective effects were associated with the SIRT1 pathway. Overall, our findings provided insights into the antioxidative stress- and anti-inflammation-related effects and mechanism of THJ in DCM. These findings may further support the clinical use of THJ. However, further studies are required to assess the constituents of THJ exhibiting efficacy.

## Data Availability

All data used to support the findings of this study are available from the corresponding author upon request.

## Abbreviations

DCM: Diabetic cardiomyopathy
T2DM: Type 2 diabetes mellitus
ROS: Reactive oxygen species
NLRP: Nucleotide-binding oligomerization domain-like receptor protein
SIRT1: Sirtuin 1
FOXOs: Forkhead transcription factors
TCM: Traditional Chinese medicine
THJ: Taohuajing
STZ: Streptozotocin
TTC: 2, 3, 5-Triphenyltetrazolium chloride
SOD: Superoxide dismutase
MDA: Malondialdehyde
GSH: Glutathione
DHE: Dihydroethidium
FBG: Fasting-blood glucose
LVFS: Left ventricular fractional shorting
LVEF: Left ventricular ejection fraction
LVEDV: Left ventricular end-diastolic volume
LVESV: Left ventricular end-systolic volume
LDH: Lactate dehydrogenase
TNF-α: Tumor necrosis factor-α
CK-MB: Creatine kinase-MB
IL-6: Interleukin-6
GSH-Px: Glutathione peroxidase
IL-1α: Interleukin-1α
TEM: Transmission electron microscopy
PVDF: Polyvinylidene difluoride
